# Self‐Reported Oral Health‐Related Quality of Life and Orofacial Esthetics Among Young Adults With Treated Dental Trauma

**DOI:** 10.1002/cre2.70068

**Published:** 2025-01-05

**Authors:** Mari Louise Odersjö, Lina Johansson, Agneta Robertson, Nina Sabel

**Affiliations:** ^1^ Clinic of Pediatric Dentistry Public Dental Service Borås Region Västra Götaland Sweden; ^2^ Clinic of Pediatric Dentistry Public Dental Service Uddevalla Region Västra Götaland Sweden; ^3^ Department of Pediatric Dentistry, Institute of Odontology at Sahlgrenska Academy University of Gothenburg Gothenburg Sweden; ^4^ Clinic of Pediatric Dentistry Public Dental Service Göteborg Region Västra Götaland Sweden

## Abstract

**Objectives:**

This study aimed to explore how young adults with a history of dental trauma and restored teeth perceive their oral health–related quality of life (OHRQoL) and orofacial esthetics, with a focus on gender‐based differences.

**Materials and Methods:**

This pilot study is a retrospective case–control study. Young adults experiencing dental trauma and consequently receiving dental treatment were asked to answer CPQ11‐14, Oral Esthetic Scale (OES), and some complimentary questions concerning the esthetics of their teeth. A control group was recruited. *t*‐Test was used to analyze the scores of CPQ_11‐14_ and OES, comparing both the study group and the control group, as well as assessing differences between the genders. Regression analysis ANOVA was used to examine the relationship between the OES questions and CPQ_11‐14_ domains.

**Results:**

The study group comprised 74 individuals (mean age = 23 years, SD 2.8), including 48 females and 26 males, with an equivalent number in the control group (mean age = 23 years, SD 3.4), with 49 females and 25 males. The mean score of the CPQ_11‐14_ was 8.8 (SD 7.2) for the study group and 8.0 (SD 6.8) for the control group. Within the emotional well‐being domain, the study group exhibited a higher mean score (3.4, SD 3.6) compared to the control group (1.5, SD 2.6) (*p* < 0.001 *t*‐test). Females in the study group scored higher in the emotional well‐being domain (4.0, SD 3.7) compared to females in the control group (1.8, SD 2.7), (*p* < 0.01 independent *t*‐test). The study group, who reported low satisfaction with their tooth color or alignment in the OES, also scored higher in the social well‐being domain of CPQ_11‐14_ (*p* < 0.026, ANOVA). This was not observed in the control group.

**Conclusions:**

Young adults with treated dental trauma trend to report a negative impact on their oral health‐related quality of life and oral esthetics, particularly females.

## 1 Introduction

Numerous worldwide studies have shown the high prevalence of traumatic dental injuries (TDIs) in children and adolescents, affecting their permanent dentition (Andreasen and Ravn 1972; Skaare and Jacobsen 2003). Frequencies ranging from 13% to 35% have been reported (Andreasen and Ravn 1972; Skaare and Jacobsen 2003; Oldin et al. 2015). Globally, over one billion people have experienced traumatic dental injuries (TDIs) (Petti, Glendor, and Andersson 2018). Children aged 8–10 years face the highest risk of sustaining dental trauma with the upper central incisors most commonly affected (Skaare and Jacobsen 2003). In the permanent dentition, hard tissue injuries prevail, i.e., uncomplicated crown fractures, often in combination with injuries of the supporting tissues (Skaare and Jacobsen 2003). The significance of TDIs, often affecting the maxillary incisors which are the most prominently visible teeth, has a profound psychological and social impact on the esthetic perception in children and young adults, thereby impacting their quality of life (Arhakis, Athanasiadou, and Vlachou 2017; Klages 2004). In summary, dental trauma is a significant health concern (Petti, Glendor, and Andersson 2018).

All TDIs are inherently distressing and stressful for the patient (Arhakis, Athanasiadou, and Vlachou 2017; Locker et al. 2002; Berger et al. 2009; Aldrigui et al. 2011; Nicolau, Marcenes, and Sheiham 2003). It is common for dental problems resulting from TDIs, i.e., pain, poor esthetics, or other physical, emotional, and psychological effects, to influence a child's Oral Health Quality of Life (OHRQoL) (Das et al. 2022; Ilma de Souza Cortes, Marcenes, and Sheiham 2002; Traebert et al. 2012). Awareness of oral and facial esthetics has grown, with TDIs impacting individuals' perception of their appearance (Robertson and Norén 1997). Schoolchildren aged 11–14 years with TDIs often report lower OHRQoL compared to their non‐injured peers (Aldrigui et al. 2011; Traebert et al. 2012; Fakhruddin et al. 2008). A Swedish study involving children aged 7–19 years, having received treatment for an acute TDI, found that 50% expressed dissatisfaction with the form and color of the dental restoration (Robertson and Norén 1997).

In a national study from Great Britain, 24% answered that they were dissatisfied with their dental appearance (Alkhatib, Holt, and Bedi 2005). Overall, there is an increasing focus on patients' requests for an esthetically pleasing dentition (Bradnock et al. 2001).

Esthetic concerns of tooth color have a higher negative impact in females compared to males, females having higher scores in the dimensions of pshychosocial impact measured via OHIP (Oral Health Impact Profile) and PIDAQ (Psychological Impact of Dental Aesthetics Questionnaire) (Kovacevic Pavicic et al. 2019). In a study concerning oral health–related quality of life (OHRQoL) and esthetics among young adults, females report having a lower OHRQoL, though no difference between genders was reported regarding satisfaction with dental esthetics (Narhi et al. 2023). Gender is seen to be an issue to be considered when it comes to esthetics and has been poorly investigated.

In a study comparing health‐related aspects across genders, women reported having lower levels of self‐rated health and functional health, compared to men (Denton, Prus, and Walters 2004). Additionally, in 5–19‐year‐olds affected by enamel dysplasia with enamel defects, the CPQ_11‐14_ scores indicated that females are more emotionally affected than males (Kohli et al. 2011). Therefore, investigation of gender‐based differences on how TDI affects OHRQoL is indicated.

Currently, there are no studies of young adults who received treatment due to TDIs during childhood, evaluating OHRQoL and orofacial esthetics, including the aspect of differences between gender. This study aimed to explore how young adults with a history of dental trauma and restored teeth perceive their OHRQoL and orofacial esthetics, with a focus on gender‐based differences.

## 2 Materials and Methods

The design is a pilot study for a retrospective case–control study performed in Sweden in 2020.

### 2.1 Patients

#### 2.1.1 Study Group

Patients born 1993–2003 and treated for TDIs of one or more permanent maxillary incisors, occurring before the age of 15 years, were included in the study. All patients sought out a dentist due to the TDI. The diagnoses of the TDI varied. Some of the participants were previously described in a prior study (Oldin et al. 2015). All dental treatments were administered at the Public Dental Service, Region Västra Götaland, Sweden. The treatment varied depending on the diagnosis of the trauma.

### 2.2 Control Group

The control group comprised patients born 1993–2003, obtained from the Public Dental Service register in Region Västra Götaland. Exclusion criteria included any self‐reported history of dental trauma.

### 2.3 Methods

#### 2.3.1 Questionnaires

Participants provided general information on age and gender.

##### 2.3.1.1 CPQ_11‐14_

Two global self‐assessment questions regarding oral health and general health status were included (Jokovic et al. 2002). Responses were categorized into five scorings, 0: *Poor*; 1: *Moderate*; 2: *Good*; 3: *Very good*; and 4: *Excellent*.

The short version of CPQ_11‐14_ consists of 16 questions reflecting the individuals OHRQoL. Participants rated the frequency of experiencing symptoms related to teeth, lips, jaws, and mouth over the past 4 weeks on a five‐score Likert scale, 0: *Never*; 1: *Rarely*; 2: *Sometimes*; 3: *Often*; or 4: *Every day/Almost every day* (Jokovic, Locker, and Guyatt 2006). A validated Swedish version of CPQ_11‐14_ with 16 questions was used (Talvilahti 2007).

Questions are sorted as a total, with the total score ranging from 0 to 64, including the four domains: oral symptoms, functional limitations, emotional well‐being and social well‐being; each on its own containing four questions categorized as a 4‐item subscale of CPQ_11‐14_, with each subscale score ranging from 0 to 16 (Jokovic, Locker, and Guyatt 2006). An additional categorization of CPQ_11‐14_ is the two‐item subscale, oral symptoms and functional limitations, forming the subscale *Symptoms/function*. Emotional well‐being and social well‐being form the other subscale, *Well‐being*. The two‐subscale scores range from 0 to 32 (Thomson et al. 2016).

##### 2.3.1.2 OES

Esthetic self‐assessment was conducted using a modified oral scale of esthetics, the Orofacial Esthetic Scale (OES). OES consists of eight questions concerning self‐perception of facial, mouth, and teeth appearance. A validated Swedish version of OES was used (Larsson, John, Nilner, Bondemark, and List 2010). Questions 1–7 collectively describe esthetics in the oro‐facial region. Question 8 represents the overall assessment of the patient's orofacial esthetics. The responses are reported on a scale from 0 (*very dissatisfied*) to 10 (*very satisfied*) (Larsson, John, Nilner, Bondemark, and List 2010; Larsson, John, Nilner, and Li 2010, 2014).

Outcome variables of the OES include the summary score and individual scores of Questions 1–7, and the individual score for Question 8. A score of 10 indicates *very satisfied*, with the proportion of responders who were *very satisfied* calculated. Additionally, a score greater than 5 is defined as *happy*.

##### 2.3.1.3 Supplementary Questions

The study groups responded to supplementary questions (Table 1). The control group only answered supplementary questions 5–8 (Table 1).

**Table 1:** Table of supplementary questions and answer options.

Supplementary questions	Response options
Have you received composite on your broken tooth?	Yes/No
If you received composite, how to you grade the restoration?	1 (*very dissatisfied*) – 10 (*very satisfied*)
Have you received a crown on your broken tooth?	Yes/No
If you received a crown, how do you grade the crown?	1 (*very dissatisfied*) – 10 (*very satisfied*)
If you compare the appearance of your teeth to your friends, how would you grade your teeth?	1 (*very dissatisfied*) – 10 (*very satisfied*)
If you compare the color of your teeth to your friends, how would you grade your teeth?	1 (*very dissatisfied*) – 10 (*very satisfied*)
Have you whitened your teeth?	Yes/No
Have you had orthodontic treatment?	Yes/No

Results of the supplementary questions are described as binominal answers. Being *happy* is defined as a score > 5, and answers of 9 or 10 are defined as *very satisfied*.

#### 2.3.2 Distribution

All questionnaires in their Swedish versions, were sent during 2020 to the study group and the control group. The responses were anonymously returned.

#### 2.3.3 Statistics

Data were analyzed using IBM SPSS statistics, version 28.0.1.0 (142). Descriptive statistics and statistical analyses of independent *t*‐test between groups and chi^2^ tests were performed comparing the dichotomized groups.

Additionally, regression analysis with ANOVA was conducted to examine the relationship between the OES questions and CPQ_11‐14_ domains.

#### 2.3.4 Ethical Considerations

All participants in the study group were informed in writing regarding the voluntary nature of their participation and provided their written consent by completing the questionnaires. The study received ethical approval from the Regional Ethical Committee in Göteborg, Sweden in October 2019 (Dnr 2019‐03306).

## 3 Results

### 3.1 Patients

#### 3.1.1 Study Group

The study group comprised 74 individuals with ages ranging between 18 and 30 years and a mean age of 23 (SD 2.8). Among them, 48 were females and 26 were males. The female participants had ages ranging from 18 to 30 years, with a mean age of 23, while the males were between 18 and 27 years, with a mean age of 23.

#### 3.1.2 Control Group

In the control group, the ages of the participants varied between 18 and 30 years, with a mean age of 23 (SD 3.4). This group included 49 females and 25 males. The female participants had a mean age of 23 years, while the males were between 18 and 30 years, with a mean age of 23.

#### 3.1.3 Descriptive Data on the CPQ_11‐14_

88% of participants reported that their oral health, including teeth, lips, jaws, and mouth, was either good, very good, or excellent.

**Figure 1:**
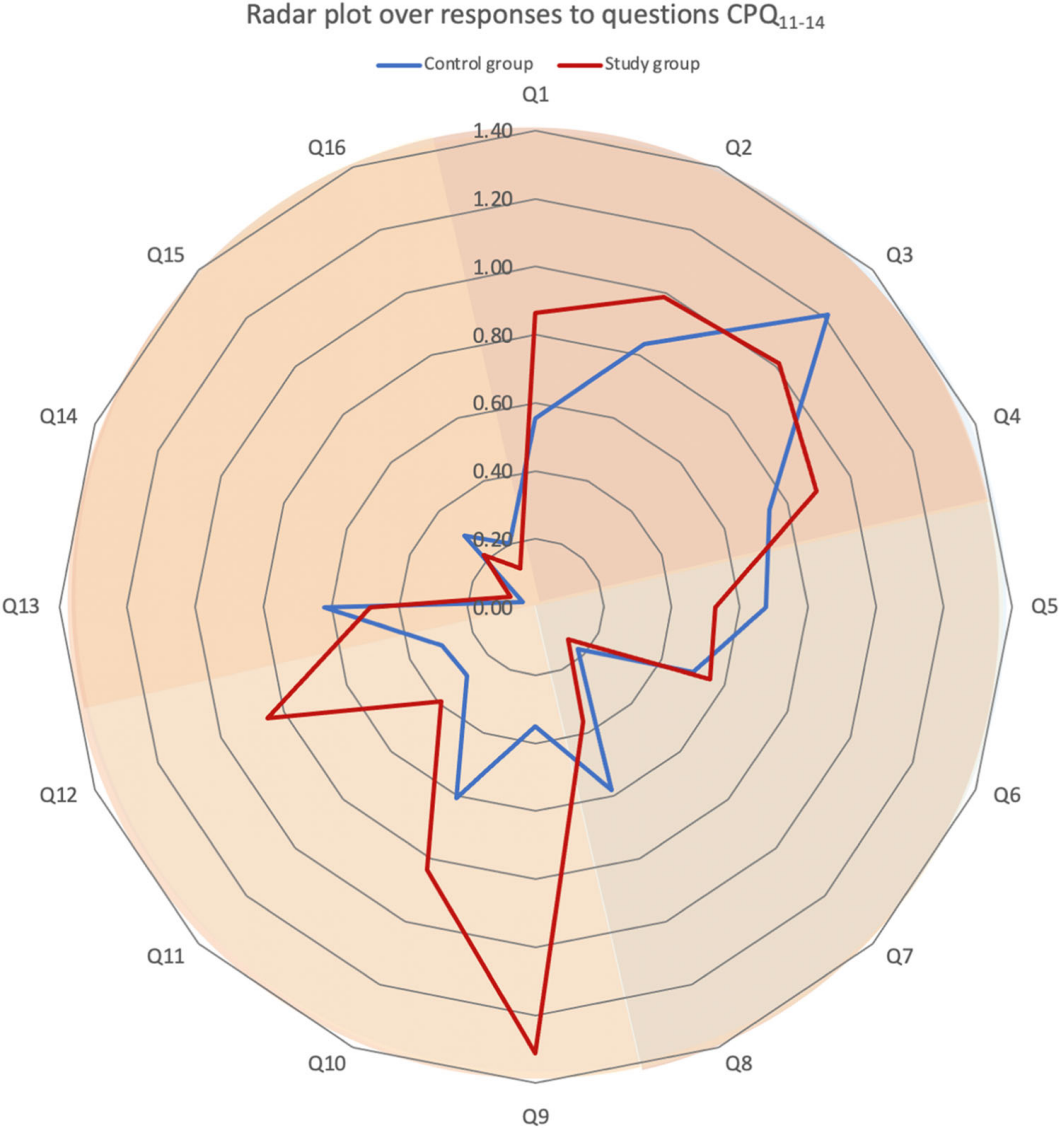
Radar plot illustrating the mean responses to the 16 questions (Q1–Q16) in CPQ_11‐14_. Mean of the scorings, 0: *Never*; 1: *Rarely*; 2: *Sometimes*; 3: *Often*; or 4: *Every day/Almost every day*. The red line represents the study group, while the blue line represents the control group. Background color indicates the four domains (Q1–Q4: Oral symptoms, Q5–Q8: Functional limitations, Q9–Q12: Emotional well‐being and Q13–Q16: social well‐being).

Concerning the response to the global question regarding oral health, 48% of the females and 35% of the males responded affirmatively. The mean scores for responses to each question (Q1–Q16) can be found in Figure 1. The mean total score of the CPQ_11‐14_ was 8.8 (SD 7.2) for the study group and 8.0 (SD 6.8) for the control group (Table 2).

**Table 2:** Descriptive data including scale range, mean score, standard deviation (SD), median, and range for the short form of the CPQ_11‐14_ in the study group and control group. A *t*‐test was conducted to analyze the scores in several comparisons: between the study group and control group, within the study group (females vs. males) and within the control group (females vs. males), and between genders across the groups (study group females vs. control group females, and study group males vs. control group males). All responders provided answers to all questions.

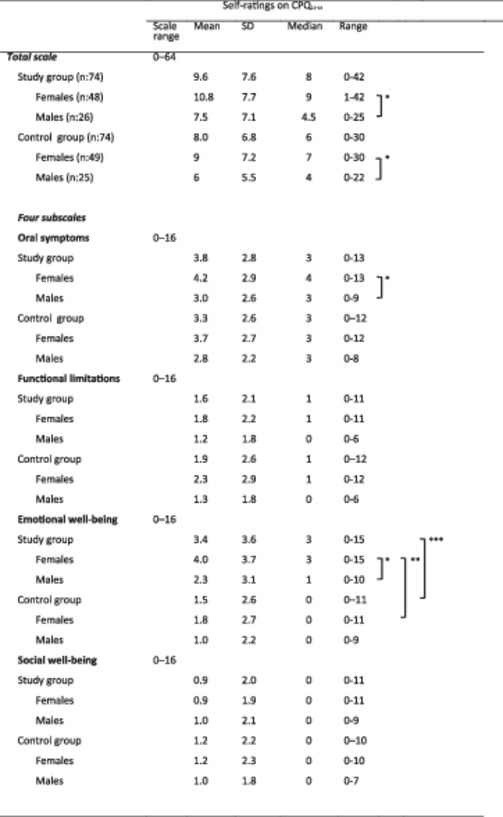

*p‐value < 0.05 independent *t*‐test analysis females compared to males (in study group and control group, respectively)

**p‐value < 0.01 independent *t*‐test analysis females with treated TDIs compared to females in the control group

***p‐value < 0.001 independent *t*‐test analysis: study group compared to control group.

Differences in total scores were observed between genders in both the study group and control group. In the study group, females scored higher (mean 10.8, SD 7.7) in total score of CPQ_11‐14_ compared to males (7.5, SD 7.1) (*p* < 0.05 independent *t*‐test). In the control group, females (mean 9, SD 7.2) scored higher than males (6, SD 5.5) (*p* < 0.05 independent *t*‐test) (Table 2).

##### 3.1.3.1 Four‐Subscale Analysis

Within the study group, the mean scores for the four‐subscale analysis ranged from 0.9 to 3.8 (Table 2). The study group exhibited a higher mean score in the emotional well‐being domain (3.4, SD 3.6) compared to the control group (1.5, SD 2.6) (*p* < 0.001, *t*‐test). Furthermore, in the study group, females scored higher in both the oral symptoms and emotional well‐being domains compared to males (*p* < 0.05 independent *t*‐test), (Table 2). Additionally, females in the study group scored higher in the emotional well‐being domain (4.0, SD 3.7) than females in the control group (1.8, SD 2.7) (*p* < 0.01, independent *t*‐test) (Table 2).

##### 3.1.3.2 Two‐Subscale Analysis

The study group exhibited a higher mean score in the well‐being domain (4.3, SD 4.8), compared to the control group (2.7, SD 4.3) (*p* < 0.05, *t*‐test).

In the study group, females scored higher (mean 6.0, SD 3.8) in the symptoms/function domain than males (mean 4.2, SD 3.8) (*p* < 0.05, independent *t*‐test). A similar difference was observed in the control group, where females scored higher (mean 5.9, SD 4.4) than males (mean 4.0, SD 2.8) (*p* < 0.05, independent *t*‐test).

Moreover, females in the study group scored higher in the well‐being domain (4.81, SD 5.1) than females in the control group (3.08, SD 4.6), (*p* < 0.05, independent *t*‐test).

#### 3.1.4 Descriptive Data on the OES

The mean and median summary of the OES scores for all participants were 39 (SD 16) and 41, respectively (Table 3). The percentage of individuals who answered *very satisfied* is presented in Table 3 for both genders in the study and control groups. A total of 59 participants provided *very satisfied* responses, with a median of 2.9 questions (ranging from 1 to 8). Notably, 28 individuals scored *very satisfied* on more than three items (11 treated TDIs and 17 without). Among the 89 individuals who did not score any *very satisfied* responses, 50 belonged to the study group, and 39 to the control group (*p* = 0.065, χ^2^), of which 59 were females and 30 were males (*p* = 0.813, χ^2^).

**Table 3:** Descriptive data for OES (Orofacial Esthetic Scale) including mean score, standard deviation (SD), median score of answers, and percentage of *very satisfied* responses from both genders in the study group and the control group. All responders provided answers to all questions.

	Self‐ratings on OES			
OES Item	Mean	SD	Median	*Very satisfied*
OES summary score (Q1–Q7)				
Trauma group (*n* = 74)	40.5	17.6	45	3%
Females (*n* = 48)	39.8	16.4	38	2%
Males (*n* = 26)	41.7	20.0	48	4%
Control group (*n *= 74)	37.4	15.2	38	3%
Females (*n *= 49)	37.4	14.9	39	2%
Males (*n* = 25)	37.2	16.2	36	4%
Face, Q1				
Trauma group	7.0	3.1	8	10%
Females	7.3	2.8	8	6%
Males	6.5	3.7	8.5	15%
Control group	6.3	3.3	8	15%
Females	6.5	3.1	8	10%
Males	6.0	3.7	7	24%
Profile, Q2				
Trauma group	6.3	3.1	7	10%
Females	6.4	2.9	7.5	6%
Males	6.0	3.6	7	15%
Control group	5.2	3.3	7	11%
Females	5.0	3.3	5	8%
Males	5.5	3.5	5	16%
Mouth, Q3				
Trauma group	6.1	3.4	8	7%
Females	6.0	3.3	7.5	6%
Males	6.3	3.6	8	8%
Control group	5.6	3.7	7	16%
Females	5.8	3.7	7	10%
Males	5.0	3.9	7	28%
Alignment, Q4				
Trauma group	5.1	3.8	5	15%
Females	4.9	3.9	3.5	15%
Males	5.4	3.9	5.5	15%
Control group	4.5	4.0	4	27%
Females	4.5	4.2	3	27%
Males	4.4	3.7	5	28%
Shape, Q5				
Trauma group	5.3	3.2	7	19%
Females	4.8	4.0	5.5	23%
Males	6.2	3.9	8	12%
Control group	5.5	3.8	6	18%
Females	5.4	3.8	6	16%
Males	5.8	3.7	8	20%
Color, Q6				
Trauma group	5.0	3.2	5	7%
Females	4.8	3.2	5	6%
Males	5.3	3.3	5	8%
Control group	5.2	3.5	8	8%
Females	5.0	3.6	5	6%
Males	5.5	3.3	7	12%
Gingiva, Q7				
Trauma group	5.8	3.7	8	22%
Females	5.6	3.8	7.5	23%
Males	6.1	3.8	8	19%
Control group	5.2	4.1	6.5	32%
Females	5.2	4.1	7	33%
Males	5.0	4.1	5	32%
Overall, Q8				
Trauma group	6.7	3.1	8	8%
Females	6.8	3.0	8	6%
Males	6.5	3.3	8	12%
Control group	6.6	3.4	8	15%
Females	6.8	3.3	8	12%
Males	6.2	3.5	8	20%

The summary score of the seven questions showed no difference between the study group (mean 40.5, SD 27.6) compared to the control group (mean 37.4, SD 15.2).

The mean score for the eight questions in the OES varied; the study group ranked *color* (Q6) (mean 5.0, SD 3.2) as the lowest satisfactory score, while *face* (Q1) (mean 7.0, SD 3.1) had the highest score. The control group ranked *alignment* (Q4) (mean 4.5, SD 4.0) as the lowest score, while the *overall* question (Q8) (mean 6.6, SD 3.4) was ranked as the highest score (Table 3).

The question related to *color* (Q6) received the lowest proportion of *very satisfied* responses in both groups (Table 3).

Conversely, in both the study and control groups, the *gingiva* (Q7) was ranked as *very satisfied* by the highest proportions, even seen in both genders (Table 3).

The question *alignment* (Q4) showed the widest range of proportions being *very satisfied* between the study group (15%) and control group (27%) (*p* = 0.069, χ^2^).

Of the individuals having treated TDIs, 77% answered that they were *happy* with their facial profile, while 55% of individuals with no experience of TDIs expressed they were *happy* with their facial profile (*p* < 0.005, χ^2^).

Among females, 75% in the study group were *happy* with their facial profile, while 51% were *happy* among females in the control group (*p* < 0.015, χ^2^).

Participants in the study group, who reported low satisfaction with their tooth color, also scored high in the social well‐being domain in CPQ_11‐14_, (*p* < 0.026, ANOVA), which was not observed in the control group.

Similarly, the study group reporting low scores in alignment in OES also scored high in social well‐being in CPQ_11‐14_ (*p* < 0.026, ANOVA), which was not found in the control group.

#### 3.1.5 Supplementary Questions

**Figure 2:**
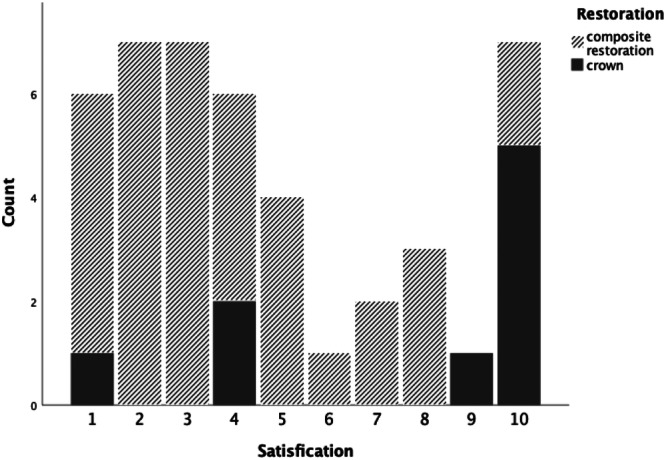
Self‐reported satisfaction scores (1 indicating *not satisfied* – 10 indicating *very satisfied*) among the study group with upper incisor restoration.

Thirty‐seven responders reported having received composite restorations, which was nearly four times as common as receiving crowns (10 individuals). The satisfaction with composite restorations varied, with seven individuals expressed being *very satisfied* (Figure 2).

Approximately two‐thirds of participants in the study group reported being *happy* with the appearance (*n *= 48) and color (*n *= 47) of their teeth compared to their friends. Correspondingly, three‐fourths of the control group reported being *happy* with the appearance (*n* = 56) and color (*n* = 54) of their teeth compared to their friends.

Nine of the participants in the study group had whitened their teeth compared to five responders in the control group.

Significantly fewer individuals in the study group (n = 28) had experience with orthodontics, compared to the control group (*n* = 43) (*p* < 0.05, χ^2^).

## 4 Discussion

To summarize, young adults having experienced dental trauma, no matter the diagnosis, and having undergone dental treatment thereafter report a negative impact on well‐being of OHRQoL in comparison to young adults with no dental trauma. Also, they express negative effects in oral esthetics. In addition, this study highlights that females tend to report a lower OHRQoL, particularly females treated for TDIs are signaling the greatest impact.

The results from this study, though small study size, are interpreted as a need for further studies including evaluation and follow‐up of the clinical esthetics after treatment due to dental trauma, to minimize consequential impact on the patients. It has previously been suggested that treatment for TDI should not solely focus on function and the consequences of the injury but should also address their impact on a person's OHRQoL, particularly in terms of emotional aspects (Antunes et al. 2020). Dental esthetics are of great concern to patients and must be carefully discussed by dentists when treating traumatic dental injuries. In aspects of clinical implication, It has previously been suggested that treatment for TDI should not solely focus on function and the consequences of the injury but should also address their impact on a person's OHRQoL, particularly in terms of emotional aspects (Antunes et al. 2020).

The group treated for trauma reported a similar total score of OHRQoL as the control group. All participants in the study group had received treatment for their traumatized teeth, which potentially explains the limited differences seen in the study and control groups concerning CPQ_11‐14_ results. Despite treatment, dental trauma significantly affects functional, esthetic, and social matters, influencing the OHRQoL of adolescents and young adults (Traebert et al. 2012). Children with fractured teeth, especially those left untreated, reported a higher impact on their social life compared to children without traumatic injuries (Fakhruddin et al. 2008). Moreover, children reported an improvement in esthetics and social interactions following treatment (Fakhruddin et al. 2008).

The instrument CPQ_11‐14_ was utilized to analyze OHRQoL. The CPQ_11‐14_ stands out as a reliable instrument to measure OHRQoL, being a consistent tool with psychometric characteristics. A limitation for this study might be that CPQ_11‐14_ was initially developed for the 11 to 14 year age group, but it has been used previously in studies for young individuals with age variations between 8 and 20 years (Berger et al. 2009; Kohli et al. 2011; Vargas et al. 2022). CPQ_11‐14_ is easily comprehensible and feasible for this young adult demographic, as supported by a previous study (Oscarson, Källestål, and Lindholm 2007). A study using OHIP and CPQ_11‐14_ observed no differences in reporting OHRQoL in 19‐year‐olds (Oscarson, Källestål, and Lindholm 2007), indicating the instruments are reliable for the age group.

Despite any limitation, this study still point out a difference in self‐reported OHRQoL seen between gender. Females report higher scores of CPQ_11‐14_, compared to males. This is supported by earlier findings, showing that females report lower OHRQoL than males at the age range of 12–18 years (Ajwa et al. 2022). The focus on the mouth during personal communication appears to be of greater importance for females than males (Armalaite et al. 2018). Females treated due to dental trauma indicate a negative impact on their emotional well‐being, compared to females in the control group. This is in congruence with a study showing that individuals aged 8–20 years, who have a history of severe dentoalveolar trauma, reporting negative effects on social and emotional well‐being aspects 1 year post‐injury (Berger et al. 2009). It is possible that this is reflected in the higher negative impact on emotional well‐being seen in the females in the study group compared to their counterparts in the control group.

The part of the study group expressing dissatisfaction with both *tooth color* and *alignment*, reported a high total score in CPQ_11‐14_, particularly in the social well‐being domain. It is essential to consider the well‐being domain, including social or and emotional domains, when analyzing the perspective of patients' reports. The questions in OES concerning *tooth color* and *alignmen*t are interpreted in this study to indicate a negative impact on OHRQoL. Dental esthetics is seen to have a direct influence on all OHRQoL domains (Klages 2004). OHRQoL influences the self‐perception of oral esthetics and vice versa. Satisfaction with *tooth color* was reported by approximately 8% of respondents, while satisfaction with *tooth alignment* was reported in less than one‐third. These results match the findings that tooth color being the primary reason for dissatisfaction (89%), followed by poor tooth alignment (24%) (Samorodnitzky‐Naveh, Geiger, and Levin 2007). Consequently, both factors appear to be important to young adults, regardless of whether they have treated TDIs or not. The current study suggests a trend in which the patients treated for trauma express less satisfaction with tooth alignment compared to the control group.

This study reveals varying levels of self‐reported satisfaction with anterior restorations, highlighting the challenge dentists face in meeting patients' estethic expectations. A German study examining patient preferences for dental crown treatment found that esthetics were of paramount importance when discussing dental replacements in the anterior region (Felgner and Henschke 2023). A study analyzing preferences for fillings from the perspectives of dentists, dental assistants, and young patients showed differences in preferences, with the patients being more sensitive to visibility and less concerned about the durability of the fillings (Espelid et al. 2006).

To summarize, when treating young individuals with anterior teeth injuries, it is crucial to make a concerted effort to satisfy both functional and esthetical views. Consequently, it is evident that further studies in this field are warranted ‐ particularly within the female population.

## 5 Conclusions

Young adults with treated dental trauma trend to report a negative impact on their oral health‐related quality of life and oral esthetics, particularly females.

## Author Contributions

*Conceptualization:* Mari Louise Odersjö and Agneta Robertson. *Data curation:* Mari Louise Odersjö, Nina Sabel, Lina Johansson, and Agneta Robertson. *Formal analysis:* Mari Louise Odersjö and Nina Sabel. *Funding acquisition:* Mari Louise Odersjö. *Investigation:* Mari Louise Odersjö and Nina Sabel. *Methodology:* Mari Louise Odersjö, Nina Sabel, Lina Johansson, and Agneta Robertson. *Project administration:* Nina Sabel. *Resources:* Mari Louise Odersjö, Nina Sabel, Lina Johansson, and Agneta Robertson. *Software:* Mari Louise Odersjö and Nina Sabel. *Supervision:* Nina Sabel. *Visualization:* Mari Louise Odersjö and Nina Sabel. *Roles/Writing–original draft:* Mari Louise Odersjö and Nina Sabel. *Writing–review and editing:* Mari Louise Odersjö, Nina Sabel, Lina Johansson, and Agneta Robertson.

## Ethics Statement

The study received ethical approval from the Regional Ethical Committee in Göteborg, Sweden in October 2019 (Dnr 2019‐03306).

## Consent

All participants in the study group were informed in writing regarding the voluntary nature of their participation and provided their written consent by completing the questionnaires.

## Conflicts of Interest

The submitting author ensures that all co‐authors confirm agreement with the final statement (see submitted material).

## Data Availability

The research data associated with a paper is available, upon request from authors.
